# Supplementary material for: Insights into structure and dynamics of extracellular domain of Toll-like receptor 5 in Cirrhinus mrigala (mrigala): A molecular dynamics simulation approach
Source: PLoS One. 2021 Jan 14;16(1):e0245358. doi: 10.1371/journal.pone.0245358 (PMC7808660; doi:10.1371/journal.pone.0245358)
Supplement: S1 File — (DOCX) [file pone.0245358.s001.docx]

**Supporting Information**

**Insights into structure and dynamics of extracellular domain of Toll-like receptor 5 in *Cirrhinus mrigala* (mrigala): A molecular dynamics simulation approach**

Ajaya Kumar Rout^1#^, Varsha Acharya^1^, Diptimayee Maharana^1^, Budheswar Dehury^2#^, Sheela Rani Udgata^3^, Rajkumar Jena^4^, Bhaskar Behera^4^, Pranaya Kumar Parida^1*^, Bijay Kumar Behera^1*^

^1^Aquatic Environmental Biotechnology & Nanotechnology (AEBN) Division, ICAR-Central Inland Fisheries Research Institute, Kolkata, West Bengal, India

^2^Department of Chemistry, Technical University of Denmark, DK-2800 Kongens Lyngby, Denmark

^3^Department of Bioinformatics, Odisha University of Agriculture and Technology, Bhubaneswar, Odisha, India

^4^Department of Biosciences and Biotechnology, Fakir Mohan University, Balasore, Odisha, India

***Corresponding authors**

Dr. Bijay Kumar Behera

**Email**: beherabk18@yahoo.co.in

Tel: +91-033-25921190 (O)

Fax: +91-033-25920388

Dr. Pranaya Kumar Parida

**Email**: pranayaparida@gmail.com

^#^Authors equally contributed to this work

**Table S1**. Inter-atomic distance of the interacting pairs forming discrete hydrogen bonds in *Cm*TLR5-flagellin and *Dr*TLR5-flagellin complexes

| ***CmTLR5-flagellin complex*** | | | ***DrTLR5-flagellin complex*** | | |
| --- | --- | --- | --- | --- | --- |
| *Interacting residues pairs* | *Avg.(nm)* | *SD* | *Interacting residues pairs* | *Avg.(nm)* | *SD* |
| Arg282(NH1)-Gly78(O) | 0.57 | 0.18 | Asn277(ND2)-Glu114(OE1) | 0.28 | 0.01 |
| Arg282(NH2)-Gly78(O) | 0.50 | 0.17 | Asn277(ND2)-Ala110(O) | 0.29 | 0.01 |
| Asn246(ND2)-Thr81(O) | 0.57 | 0.18 | Lys242(NZ)-Gln96(OE1) | 0.30 | 0.05 |
| Lys163(NZ)-Ans197(OD1) | 0.47 | 0.16 | Gly270(O)-Arg89(NH1) | 0.28 | 0.01 |
| Tyr248(O)-Arg89(NH1) | 0.28 | 0.01 | Tyr267(O)-Arg89(NH1) | 0.27 | 0.01 |
| Gly251(O)-Arg89(NH1) | 0.29 | 0.02 | Ser271(O)-Arg89(NH2) | 0.30 | 0.02 |
| Ser252(O)-Arg89(NH2) | 0.29 | 0.01 | Arg37(NH1)-Asn219(OD1) | 0.29 | 0.02 |
| His256(NE2)-Glu114(OE1) | 0.38 | 0.07 | Gln80(NE2)-Ala208(O) | 0.29 | 0.01 |
| Asn258(OD1)-Gln96(NE2) | 0.34 | 0.09 | Gln80(NE2)-Asn211(OD1) | 0.29 | 0.01 |
| Ser249(OG)-Glu92(OE1) | 0.37 | 0.12 | Glu79(OE1)-Asn211(ND2) | 0.28 | 0.01 |
| Asp34(OD2)-His215(NE2) | 0.35 | 0.15 | Lys301(NZ)-Glu82(OE2) | 0.39 | 0.15 |
| Gln110(NE2)-Ala204(O) | 0.34 | 0.08 |  | - | - |
| Glu136(OE1)-Arg203(NH2) | 0.35 | 0.12 |  | - | - |
| Glu136(OE2)-Arg203(NH1) | 0.39 | 0.14 |  | - | - |
| Thr3(OG1)-Asn218(O) | 0.55 | 0.19 |  | - | - |
| Gly299(O)-Gln88(NE2) | 0.34 | 0.11 |  | - | - |
| Thr189(OG1)-Gln88(NE2) | 0.36 | 0.10 |  | - | - |

^*^Avg: Average; SD: Standard Deviation


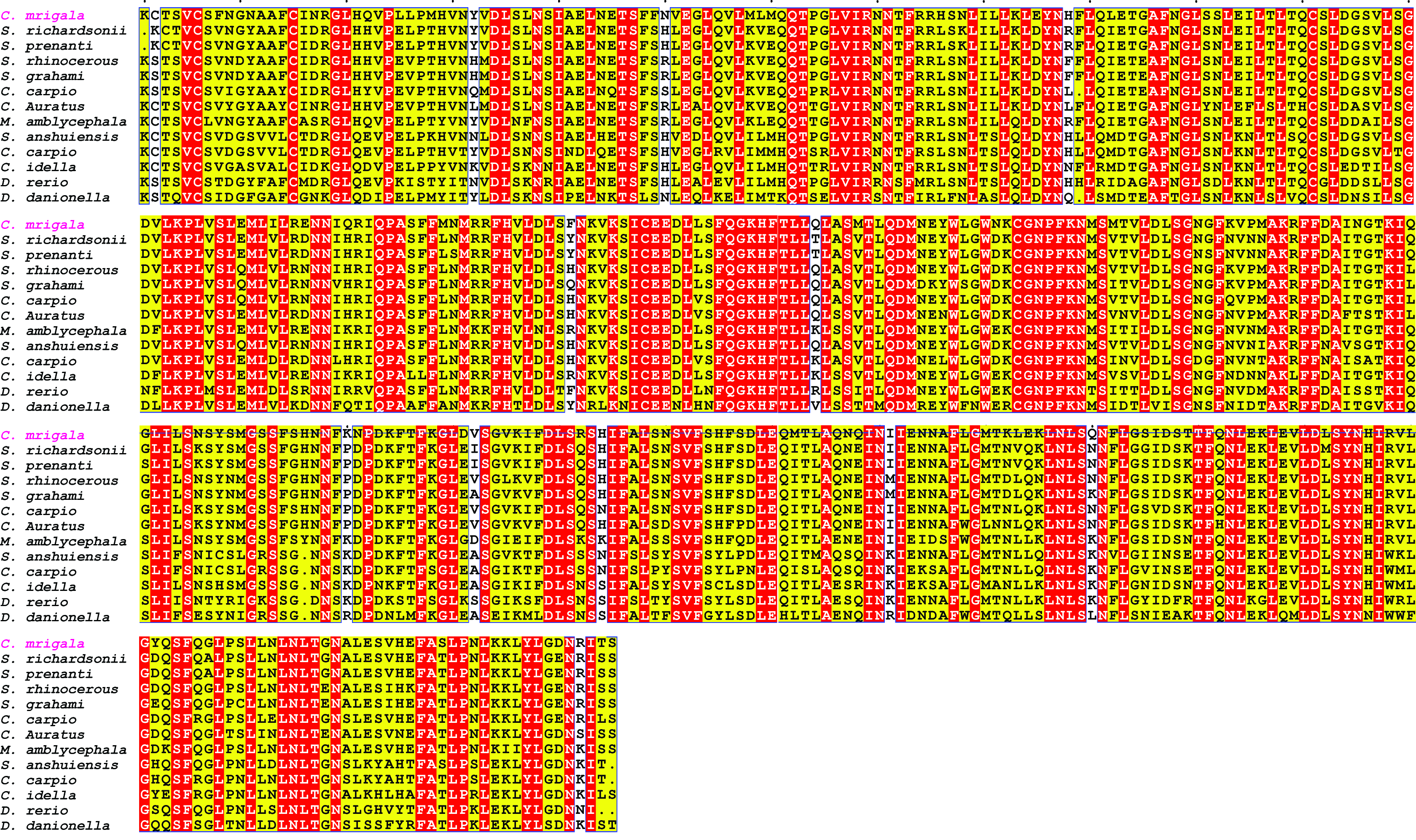


**S1 Fig**. Multiple sequence alignment of extra cellular domain (ECD) of TLR5 from *Cirrhinus mrigala* with its close homologs. The homologous sequences were retrieved from BLAST search of the TLR5-ECD from mrigala against non-redundant (nr) database of NCBI. The strongly conserved regions are highlighted in red squared boxes with white font, the conserved regions in yellow shade with black font and the variable regions without any shared with black fonts.


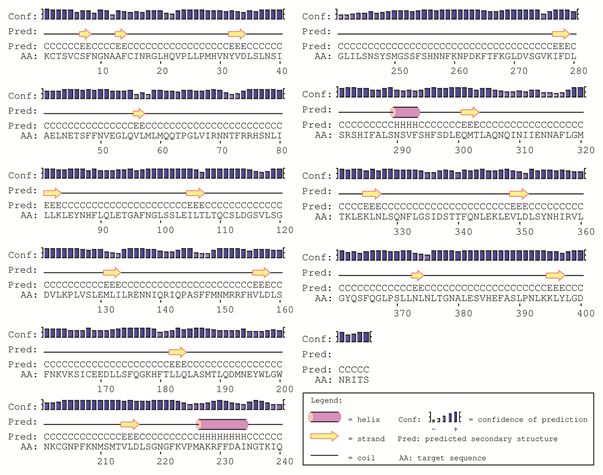


**S2 Fig.** Predicted secondary structure of the ECD of the *Cm*TLR5 using PSIPRED.


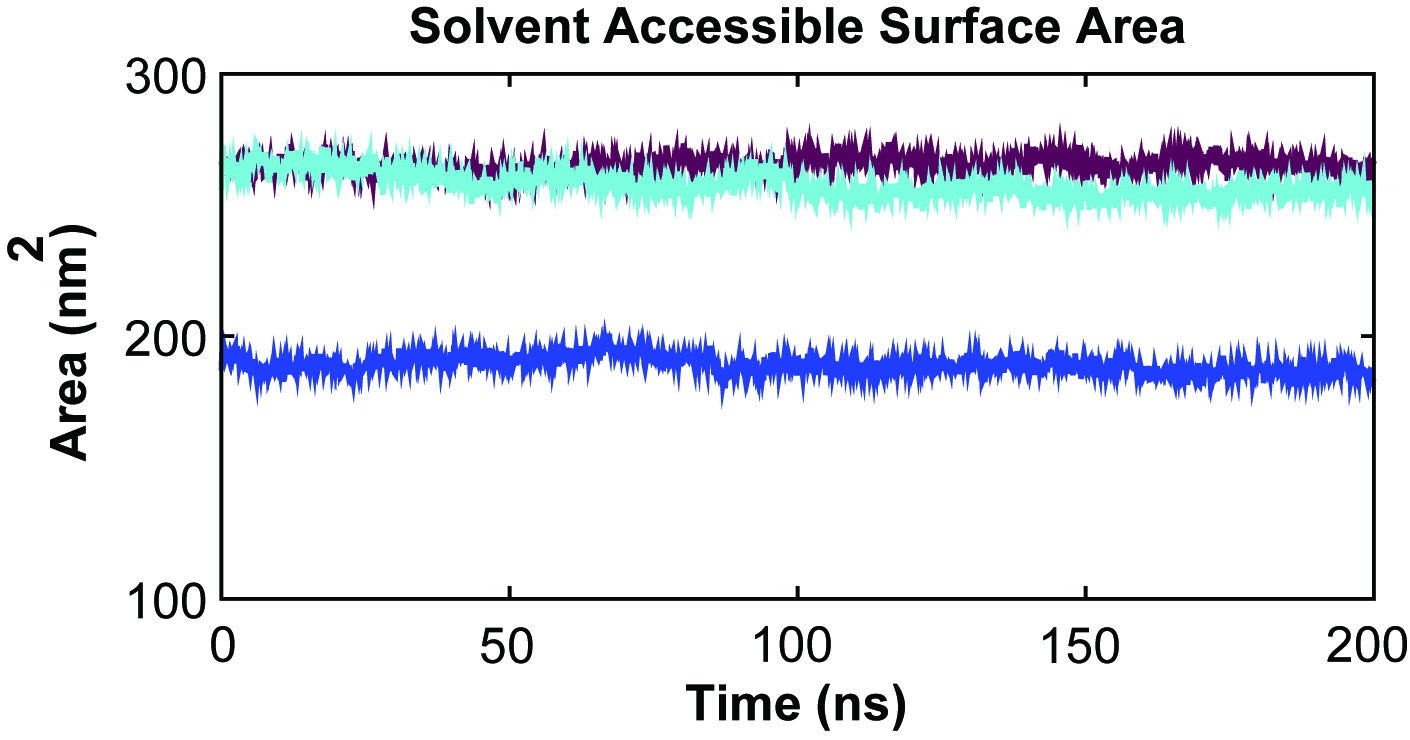


**S3 Fig**. Solvent accessible surface area (SASA) of the *Cm*TLR5 (alone: blue), *Cm*TLR5-flaggelin (maroon) and *Dr*TLR5-flagellin (cyan) complex systems during 200 ns MD.


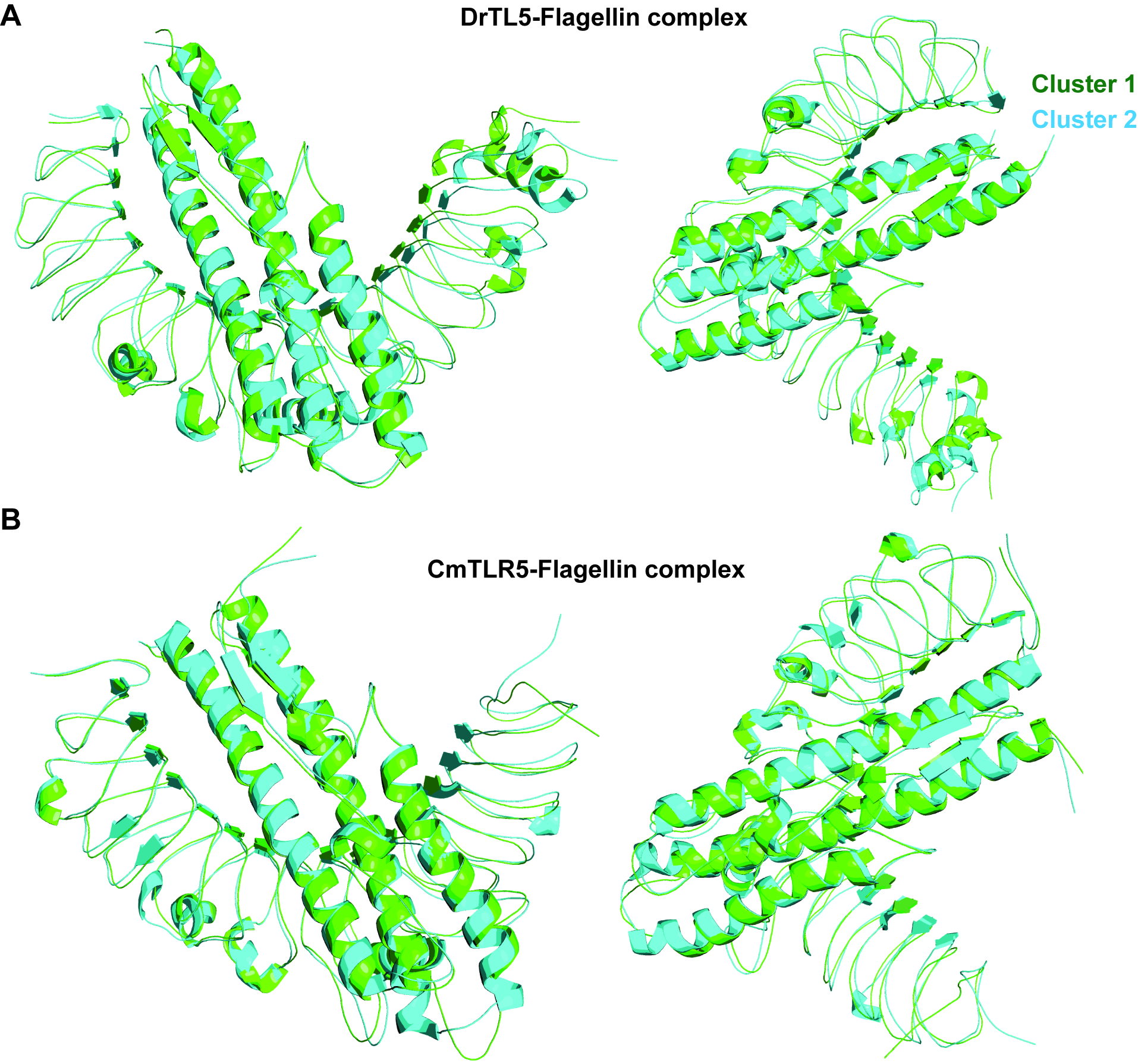


**S4 Fig**. Structural superimposed view of the top two cluster representatives from *Dr*TLR5-flagellin (**A**) and *Cm*TLR5-flagellin complexes (**B**).

**
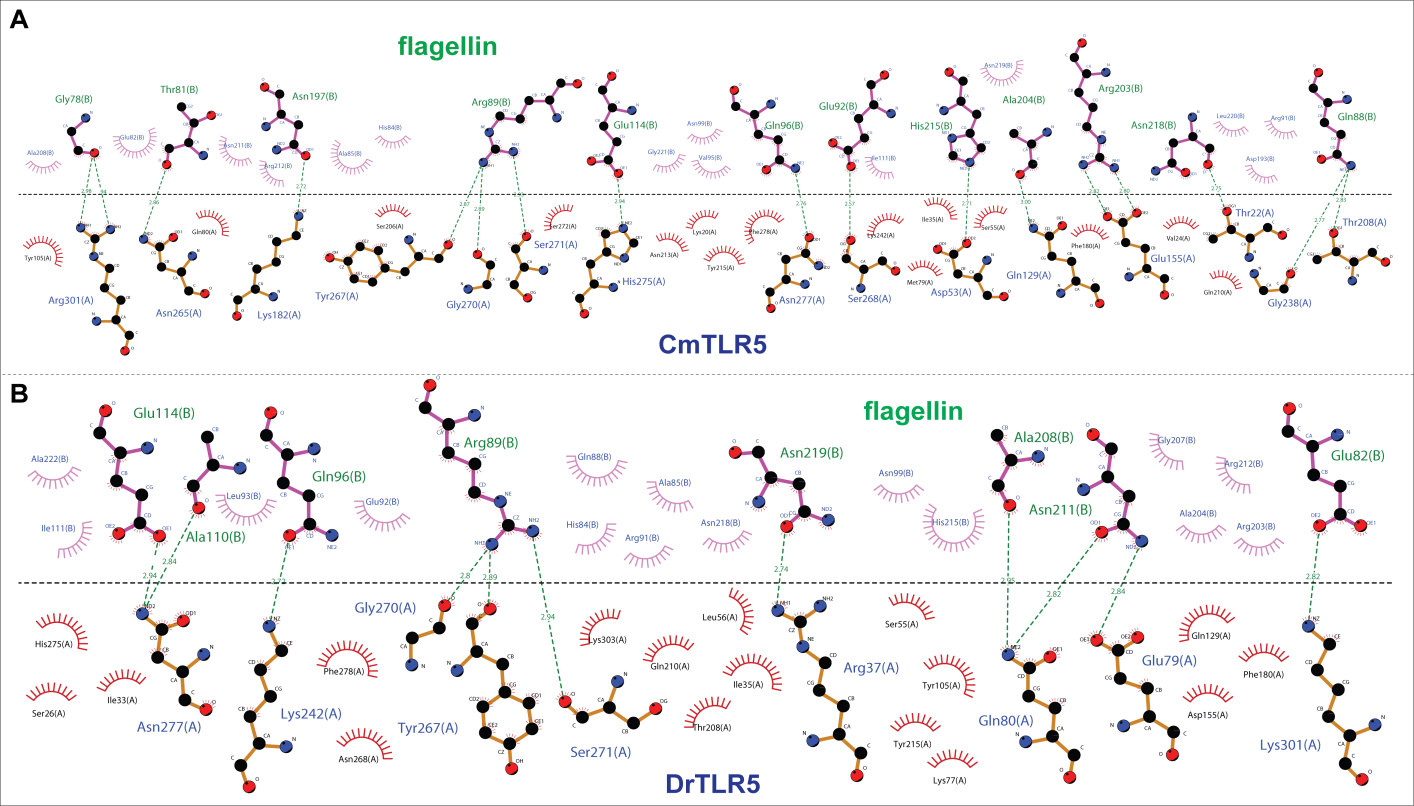
**

**S5 Fig.** Intermolecular contact analysis of flagellin and TLR5 displaying various non-bonded interactions**. (A)** Contact analysis of *Cm*TLR5-flagellin complex obtained after MD. **(B)** Contact analysis of *Dr*TLR5-flagellin complex obtained after MD. The hydrogen bonds are shown in dashed lines while the interacting residues are shown in ball and stick representation. The other hydrophobic contacts are shown in semi-circles.


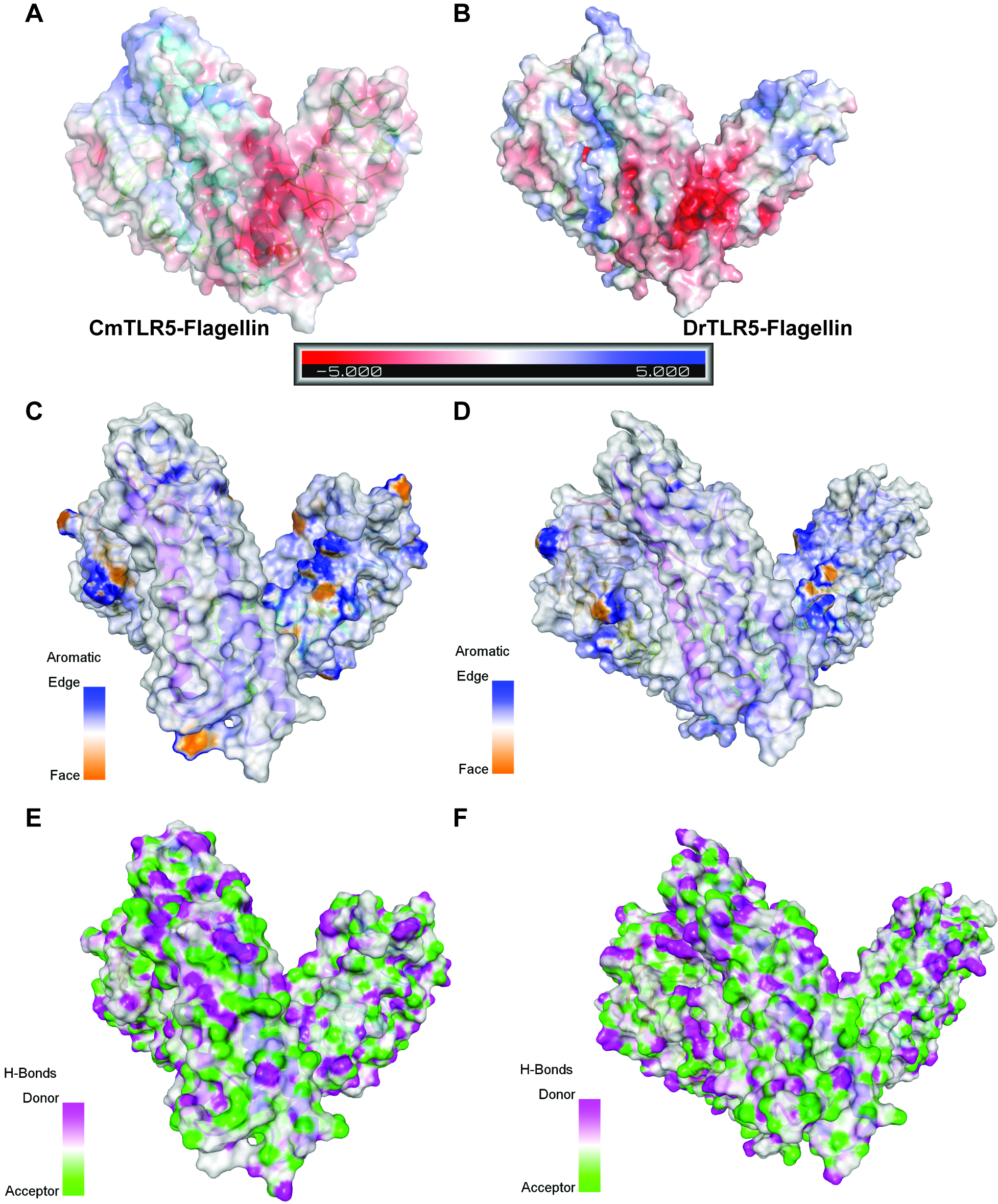


**S6 Fig.** Electrostatic surface potential map of the MD simulated *Cm*TLR5-flagellin (**A**) and *Dr*TLR5-flagellin complexes computed using APBS plug-in in PyMOL (**B**). **(C)** Aromatic surface/edge surface of the *Cm*TLR5-flagellin complex (**D**) Aromatic surface/edge surface of the *Dr*TLR5-flagellin complexes (**E**) Donor-acceptor surface displaying the H-bonds in *Cm*TLR5-flagellin complex (**F**) Donor-acceptor surface displaying the H-bonds in *Dr*TLR5-flagellin complex
