# Supplementary material for: Insights into structure and dynamics of extracellular domain of Toll-like receptor 5 in Cirrhinus mrigala (mrigala): A molecular dynamics simulation approach
Source: PLoS One. 2021 Jan 14;16(1):e0245358. doi: 10.1371/journal.pone.0245358 (PMC7808660; doi:10.1371/journal.pone.0245358)
Supplement: Attachment — Submitted filename: Response to reviewers.docx [file pone.0245358.s002.docx]

**Response to Reviewer’s**

*We would to like thank the peer reviewers for positive their feedback and valuable suggestion on our MS (PONE-20-36156). We have tried our best to address each comment raised by the reviewers. Please find the point by point response to each comment below.*

**Reviewer #1**

**Comment**

The authors should include the latest updates on TLR5 from fishes as well as other homologs.

**Response**

*As per the reviewer’s suggestion, we have performed a multiple sequence alignment of the ECD of TLR5 from mrigala will its close homologs obtained after BLAST against NR database of NCBI (****S1Fig****).*

**Comment**

It would be suggested to authors to calculate and report the distance (minimum distance) among the important interacting pairs of residues in both CmTLR5-flagellin and DrTLR5-flagellin trajectories.

**Response**

*Thank you so much for raising this important question. We have computed the distance between the interacting pairs (the Hydrogen-bond forming residues in TLR5 and Flagellin) in both the complexes using the MD trajectories (as shown in* ***Figure 9 and 10****). We have also included a paragraph describing the important interacting pairs in both the complexes (****Page no: 27*** *and paragraph:* ***Interaction of Flagellin with TLR5****) and the average distance has been present in* ***Table S1****.*

**Comment**

The discussion section needs improvement.

**Response**

*The discussion section of the MS has been revised as per the suggestion.*

**Reviewer #2**

**Comment** 1

The authors should include a multiple sequence alignment of ECD from TLR5 including mrigala with its close homologs displaying the conserved and variable regions.

**Response**

*As per the reviewer’s suggestion, we have included a multiple sequence alignment of the ECD from TLR5 of mrigala with its close homologs. The alignment displays the most conserved and variable region in the ECD of TLR5 from diverse species (****S1 Fig****).*

**Comment**

The Introduction section should be modified and the latest references need to be included.

**Response**

*The Introduction has been revised keeping the suggestion of the peer reviewer with latest research articles.*

**Comment**

The authors may represent the distance analysis of both DrTLR5-flagellin and CmTLR5-flagellin.

**Response**

*As per the reviewer recommendation we have performed distance analysis of both the complexes (as summarized in* ***Figure 9 & 10 and Table S1****).*

**Comment** 4

The authors may describe elaborately Figure 1 - the phylogeny tree

**Response**

*In the revised MS, we have discussed the findings from phylogenetic analysis as shown in Figure 1.*

**Comment**

The authors should also discuss the findings of their study with the existing reports

**Response**

*The discussion part has been revised as suggested by the Reviewer and incorporated in the manuscript.*

**Comment**

The conclusion has to be more concise.

**Response**

*As per the reviewers comment we have revised the conclusion section in the manuscript.*
